# Seroprevalence of Asian Lineage Chikungunya Virus Infection on Saint Martin Island, 7 Months after the 2013 Emergence

**DOI:** 10.4269/ajtmh.15-0308

**Published:** 2016-02-03

**Authors:** Noellie Gay, Dominique Rousset, Patricia Huc, Séverine Matheus, Martine Ledrans, Jacques Rosine, Sylvie Cassadou, Harold Noël

**Affiliations:** Regional Office of the French National Institute for Public Health Surveillance, Martinique, French West Indies; Institut Pasteur de la Guyane, Cayenne, French Guiana; Laboratoire Saint Martin Biologie, Saint Martin, French West Indies; French National Institute for Public Health Surveillance, Saint Maurice, France

## Abstract

At the end of 2013, chikungunya virus (CHIKV) emerged in Saint Martin Island, Caribbean. The Asian lineage was identified. Seven months after this introduction, the seroprevalence was 16.9% in the population of Saint Martin and 39.0% of infections remained asymptomatic. This moderate attack rate and the apparent limited size of the outbreak in Saint Martin could be explained by control measures involved to lower the exposure of the inhabitants. Other drivers such as climatic factors and population genetic factors should be explored. The substantial rate of asymptomatic infections recorded points to a potential source of infection that can both spread in new geographic areas and maintain an inconspicuous endemic circulation in the Americas.

Chikungunya virus (CHIKV) is a virus of the family *Togaviridae*, genus *Alphavirus*, transmitted by *Aedes* mosquitoes, first isolated in Tanzania in 1953.^1^ The burden of the disease is related to persistent arthralgia that sometimes outlasts the initial characteristic triad: fever, arthritis, and rash.^2^ CHIKV strains can be divided into three genetic lineages: west African, east/central/south African (ECSA), and Asian lineage.^3^ Over the last decade, the ECSA lineage became prevalent worldwide causing outbreaks in Europe, Africa, Indian Ocean, and south Asia.^4^

Saint Martin Island is divided into two parts: in the north, the French overseas territory of Saint Martin (∼36,000 inhabitants) and in the south, Sint Maarten (∼40,000), country of the kingdom of the Netherlands. The first cases of CHIKV infection in the Americas were identified in Saint Martin in November 2013; the Asian lineage was involved.^5^ The spread of CHIKV from human to human by the widely distributed vector *Aedes aegypti* quickly evolved into an outbreak. By February 2014, weekly clinical cases of CHIKV infections diagnosed by general practitioners peaked at 226 (Figure 1

**Figure 1.**
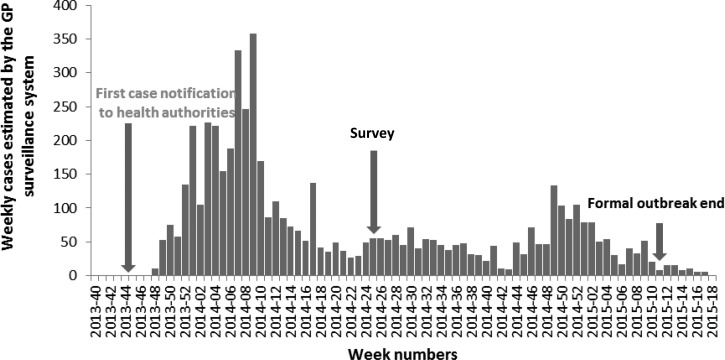
Number of weekly incident cases of chikungunya reported by the general practitioner surveillance system, Saint Martin.

). Since March, CHIKV circulation decreased in Saint Martin to a weekly average of 42 clinical cases.

In early July, with the beginning of the wet season, we expected an increased activity of *A. aegypti* vector mosquitoes. To anticipate the dynamics of chikungunya in Saint Martin and inform decision making, we conducted a serosurvey to assess the level of herd immunity and the proportion of asymptomatic infections.

For this serosurvey, we constituted a convenience sample, taking advantage of the sole laboratory of Saint Martin. Between July 3 and 8, 2014 (Figure 1), all individuals attending the laboratory for any type of biological analysis were offered a serological test for CHIKV. Only people living in Saint Martin for over 6 months were included. Participants or their legal guardian signed an informed consent and completed a questionnaire collecting information on gender, age, and possibly symptoms of CHIKV infection (joint pain and fever) during the last 6 months. A 5 mL sample of venous blood was collected from adults and 2 mL for children (those less than 6 months of age were excluded). The Advisory Ethical Committee of Paris and the French Data Protection Authority approved the study. Sera were collected and kept at −20°C for 1–6 days before being sent in dry ice to the regional French National Reference Center for Arboviruses, Institut Pasteur de Guyane. Both IgM and IgG anti-CHIKV-specific antibodies were screened in sera using an “in-house” enzyme-linked immunosorbent assay (IgM antibody capture ELISA and ELISA, respectively) as described by Talarmin and others.^6^ If IgM or IgG were detected, the serological test was considered positive.

We performed statistical analysis using STATA version 10 (www.stata.com). After a direct standardization by gender and age using 2010 Census data of the French National Institute of Statistics and Economic Studies, we estimated the seroprevalence and the proportion of asymptomatic infections in the population.

During the survey, 203 individuals were included (participation rate = 93%) and 42 (20.69%) tested positive for anti-CHIKV antibodies (19 for IgM, 36 for IgG, and 13 for both). Table 1 presents standardized estimates of the seroprevalence in Saint Martin inhabitants. We estimated that, in July 2014, the seroprevalence of CHIKV infection in the general population of Saint Martin was 16.9% (95% confidence interval [CI] = 11.6–22.1%]. Among the 42 seropositive individuals, 17 did not report any CHIKV-related symptoms. Thus, the infection remained asymptomatic for 39.0% (95% CI = 23.9–54.1%) of infected individuals in the population.

This is the first CHIKV serosurvey in the Western Hemisphere since the 2013 emergence in the Caribbean. Seven months after this emergence, we estimated that 16.9% of Saint Martin inhabitants were infected. This moderated herd immunity indicated an intermediate stage of the outbreak in July 2014.

Although a direct standardization by gender and age has been applied to limit the selection bias in patient recruitment, an underestimation of CHIKV seroprevalence in population could not be excluded. Access to health care could be easier for people coming to the laboratory than in the general population but should be unlikely as French regulation ensures a broad access to health care. Even if the geographic distribution of the individuals coming to the laboratory was not considered, CHIKV was widespread in all districts of Saint Martin.

CHIKV circulated in the whole island; the seroprevalence should be comparable for Sint Maarten and Saint Martin because of the small size of the Island and the lack of physical or geographical separation between both countries facilitating population mixing. However, no surveillance data are available for Sint Maarten, hence the attack rate cannot be directly extrapolated for Sint Maarten.

Seroprevalence reported in other surveys varied between 10.2% and 75% (Table 2). Except for the 2007 outbreak in Italy,^8^ the seroprevalence observed in Saint Martin in July 2014 was the lowest recorded. This moderate attack rate was pointing the persistence of the viral circulation in the following months (confirmed by the surveillance data; Figure 1). However, comparison with other surveys should be cautious because data were collected in different settings and at different times throughout the course of the outbreaks (elapsed time between emergence and serosurvey often missing).

The reason explaining the moderate seroprevalence in Saint Martin may lie in the local determinants of transmission of the Asian lineage with regard to the vectors, environment, and human population.

First, transmission efficiency of the local vector, *Ae. aegypti*, for the Asian lineage should be considered. Remarkably, outbreaks of Asian lineage associated with *Ae. aegypti* were limited in New Caledonia (33 and 30 autochthonous cases, in 2011 and 2013, respectively)^15^,^16^ and in Malaysia (1998 and 2006, respectively)^9^ whereas the 2009 ECSA lineage had a nationwide diffusion.^17^ However, a recent study has demonstrated that *Ae. aegypti* populations from Saint Martin were well adapted to CHIKV and transmitted efficiently both Asian and ECSA lineages.^18^

Different climatic factors (e.g., ambient temperature, daily fluctuation of temperature, and pluviometry) could have driven outbreak courses resulting in different attack rates. Recently, a model explaining autochthonous transmission of CHIKV in the Americas with climatic drivers (mean temperature and precipitations) was created.^19^ For instance, the potential link between the highest rainfall level of October and November (http://www.meteofrance.com/) and the peak of cases observed in December and January (2013 and 2014; Figure 1) should be investigated.

Besides, the moderate seroprevalence observed in Saint Martin could be indicative of the efficiency of control measures applied promptly after the emergence and throughout the outbreak (i.e., insecticide treatment, breeding sites destruction, and recommendations of personal protection against mosquitoes). Sensitized by the CHIKV outbreak in La Reunion, the French public health authorities set up in 2013 a preparedness and response plan for CHIKV introduction in Saint Martin,^20^ which may have slow down the dissemination of the virus in the population.

Our results indicated that 40.5% of the infected people did not reported chikungunya-like symptoms within the 6 months preceding the study (39.0% of asymptomatic cases in the general population of Saint Martin). Although a recall bias cannot be excluded, severe arthralgias caused by CHIKV are usually memorable.

The proportion of asymptomatic infections reported in other surveys was overall substantially lower than the proportion obtained in Saint Martin (Table 2). However, high rate of asymptomatic infections were recorded with ECSA lineages in Thailand and Kenya (47.1% and 45.1%, respectively).^12^,^14^

Manimunda and others suggested an association between the overall seroprevalence in a population and the proportion of unapparent infection. Indeed intensity of transmission in a population, loosely approximated by the attack rate, could be inversely associated to the proportion of unapparent infection.^21^

The 16.9% seroprevalence estimated gives a clearer picture of susceptible people who could still be naive (83.1%) in July 2014. Because of that large part of naive population and frequent arrival of susceptible among tourists, control efforts should be pursued. The strengthening of the viral circulation observed in December 2014 has indicated the possibility of other epidemic waves. Moreover, our study highlighted a substantial rate of asymptomatic infections that may play a significant role in maintaining the transmission.^22^ A low endemic circulation of the Asian lineage in the Americas could not be excluded as observed in Malaysia.^9^

Asian CHIKV lineage is currently disseminating in the Americas^23^ and ECSA have emerged in Brazil.^24^ CHIKV outbreaks caused by ECSA and Asian lineages could become common wherever competent vectors, *Ae. aegypti* or *Aedes albopictus*, are established in the Western Hemisphere. Our study highlighted the need of a preparedness plan to mitigate the dissemination of the CHIKV. A particular attention should be paid to the substantial rate of asymptomatic infections recorded. Even if difficult to registered by the epidemiological surveillance system, asympytomatic individuals are still a potential source of infection that can spread in new geographic area.^14^ Finally, further studies are needed in the Americas to monitor closely for each CHIKV lineage: the spread, the related proportion of unapparent infection, and the transmission efficiency by the local vector mosquitoes.

## Figures and Tables

**Table 1:** Seroprevalence of IgG and/or IgM against CHIKV in Saint Martin, July 2014

	Sample size *n* (%)	Population of Saint Martin *N* (%)	Standardized seroprevalence (%)*
Age group			
6 months to 29 years	37 (18.2)	18,197 (0.49)	14.8 (3.1–26.5)
30–44 years	55 (27.1)	9,070 (0.25)	11.7 (3.0–20.4)
45–59 years	66 (32.5)	6,726 (0.18)	21.6 (11.4–31.7)
≥ 60 years	45 (22.2)	2,983 (0.08)	34.5 (20.5–48.7)
Gender			
Men	74 (36.5)	17,519 (0.47)	18.6 (9.5–27.8)
Women	129 (63.5)	19,461 (0.53)	15.2 (8.9–21.5)
Total	203 (100)	36,980	16.9 (11.6–22.1)

CHIKV = chikungunya virus.

* Sex and age adjusted.

**Table 2:** CHIKV seroprevalence and asymptomatic rates reported in other serosurveys

Author	Date of completion	Location	Virus lineage	Primary vector	Attack rate (*N*)	Proportion of asymptomatic cases	Population sampling
Kumar and others^7^	2007 (during the outbreak)	Kerala, India	IOL	*Aedes albopictus*	55.8% (259/381)	3.8% (10/260)	Systematic clustered
Moro and others^8^	2007 (3–5 months post-outbreak)	Emilia-Romagna, Italy	IOL	*Ae. albopictus*	10.2% (33/325)	18.2% (6/33)	Systematic random
Ayu and others^9^	2007 (1 year post-outbreak)	Bagan Panchor, Malaysia	Asian	*Aedes* spp.	55.6% (40/72)	17.5% (7/40)	Systematic clustered
Sissoko and others^10^	2007 (post-outbreak)	Mayotte	IOL	*Ae. albopictus*	38.1% (440/1,154)	27.7% (122/440)	Multistage cluster
				*Aedes aegypti*			
Gérardin and others^11^	2006 (during outbreak)	La Réunion	IOL	*Ae. albopictus*	18.2% (162/888)	Not estimated	Stored sera of pregnant women
Gérardin and others^11^	2006 (post-outbreak)	La Réunion	IOL	*Ae. albopictus*	38.2% (weighted estimate: 967/2,442)	16.7% (162/967)	Systematic random
Sergon and others^12^	2004 (9 weeks after the peak of the outbreak)	Lamu Island, Kenya	ECSA	*Aedes* spp.	75% (215/288)	45.1% (118/215)	Systematic proportional to size of census unit
Sergon and others^13^	2005 (at the peak of the outbreak)	Grande Comore Island, Comoros	IOL	*Ae. aegypti*	63.1% (209/331)	14.3% (30/209)	Systematic multistage
Nakkhara and others^14^	2011 (2 years after the beginning of the outbreak	Phatthalung, Thailand	IOL	*Ae. albopictus*	61.9% (314/507)	47.1% (148/314)	Systematic (whole village)
				*Ae. aegypti*			

CHIKV = chikungunya virus; ECSA = east/central/south African; IOL = Indian ocean lineage.
